# Pneumococcal Carriage and Disease in Adults in England, 2011–2019: The Importance of Adults as a Reservoir for Pneumococcus in Communities

**DOI:** 10.1093/infdis/jiae351

**Published:** 2024-07-16

**Authors:** Dima El Safadi, Lisa Hitchins, Ashleigh Howard, Parvinder Aley, Jaclyn Bowman, Marta Bertran, Andrea Collins, Rachel Colin-Jones, Filora Elterish, Norman K Fry, Stephen S Gordon, Kate Gould, Jason Hinds, Emilie Horn, Angela Hyder-Wright, Rama Kandasamy, Shamez Ladhani, David Litt, Elena Mitsi, Annabel Murphy, Andrew J Pollard, Emma Plested, Sherin Pojar, Helen Ratcliffe, Maria C Robertson, Hannah Robinson, Matthew D Snape, Carla Solórzano, Merryn Voysey, Elizabeth Begier, Julie Catusse, Maria Lahuerta, Christian Theilacker, Bradford D Gessner, Karen S Tiley, Daniela M Ferreira

**Affiliations:** Department of Clinical Sciences, Liverpool School of Tropical Medicine; Department of Clinical Sciences, Liverpool School of Tropical Medicine; Department of Clinical Sciences, Liverpool School of Tropical Medicine; Oxford Vaccine Group, Department of Paediatrics, University of Oxford; National Institute for Health and Care Research, Oxford Biomedical Research Centre; Oxford Vaccine Group, Department of Paediatrics, University of Oxford; Immunisations and Vaccine Preventable Diseases, UK Health Security Agency, London; Department of Clinical Sciences, Liverpool School of Tropical Medicine; Oxford Vaccine Group, Department of Paediatrics, University of Oxford; Oxford Vaccine Group, Department of Paediatrics, University of Oxford; Immunisations and Vaccine Preventable Diseases, UK Health Security Agency, London; Department of Clinical Sciences, Liverpool School of Tropical Medicine; Institute for Infection and Immunity, St George's University, London; BUGS Bioscience, London Bioscience Innovation Centre; Institute for Infection and Immunity, St George's University, London; BUGS Bioscience, London Bioscience Innovation Centre; Department of Clinical Sciences, Liverpool School of Tropical Medicine; Department of Clinical Sciences, Liverpool School of Tropical Medicine; Oxford Vaccine Group, Department of Paediatrics, University of Oxford; Immunisations and Vaccine Preventable Diseases, UK Health Security Agency, London; Immunisations and Vaccine Preventable Diseases, UK Health Security Agency, London; Department of Clinical Sciences, Liverpool School of Tropical Medicine; Oxford Vaccine Group, Department of Paediatrics, University of Oxford; Department of Clinical Sciences, Liverpool School of Tropical Medicine; Oxford Vaccine Group, Department of Paediatrics, University of Oxford; Oxford Vaccine Group, Department of Paediatrics, University of Oxford; National Institute for Health and Care Research, Oxford Biomedical Research Centre; National Institute for Health Research Clinical Research Network, Thames Valley and South Midlands, Oxford; Department of Clinical Sciences, Liverpool School of Tropical Medicine; Oxford Vaccine Group, Department of Paediatrics, University of Oxford; Department of Clinical Sciences, Liverpool School of Tropical Medicine; Oxford Vaccine Group, Department of Paediatrics, University of Oxford; National Institute for Health and Care Research, Oxford Biomedical Research Centre; National Institute for Health Research Clinical Research Network, Thames Valley and South Midlands, Oxford; Oxford Vaccine Group, Department of Paediatrics, University of Oxford; National Institute for Health and Care Research, Oxford Biomedical Research Centre; Department of Clinical Sciences, Liverpool School of Tropical Medicine; Oxford Vaccine Group, Department of Paediatrics, University of Oxford; Oxford Vaccine Group, Department of Paediatrics, University of Oxford; Pfizer Vaccines, Pfizer Inc, Collegeville, Pennsylvania; Pfizer Vaccines, Pfizer Inc, Collegeville, Pennsylvania; Pfizer Vaccines, Pfizer Inc, Collegeville, Pennsylvania; Pfizer Vaccines, Pfizer Inc, Collegeville, Pennsylvania; Pfizer Vaccines, Pfizer Inc, Collegeville, Pennsylvania; Oxford Vaccine Group, Department of Paediatrics, University of Oxford; Department of Clinical Sciences, Liverpool School of Tropical Medicine; Oxford Vaccine Group, Department of Paediatrics, University of Oxford

**Keywords:** pneumococcal, carriage, transmission, invasive pneumococcal disease, adults

## Abstract

**Background:**

Pneumococcal carriage in healthy adults and its relationship to invasive pneumococcal disease (IPD) is not well understood.

**Methods:**

Nasal wash samples from adults without close contact with young children (Liverpool, UK), 2011–2019, were cultured, and culture-negative samples tested by polymerase chain reaction (PCR). Pneumococcal carriage in adults 18–44 years was compared with carriage among pneumococcal conjugate vaccine–vaccinated children aged 13–48 months (nasopharyngeal swabs, Thames Valley, UK) and national IPD data, 2014–2019. Age group–specific serotype invasiveness was calculated and used with national IPD data to estimate carriage serotype distributions for ≥65 years.

**Results:**

Overall, 98 isolates (97 carriers) were identified (3 solely by PCR) from 1631 ≥18 years adults (standardized carriage prevalence 6.4%). Despite different carriage and IPD serotype distributions between adults and children, serotype invasiveness was highly correlated (*R* = 0.9). Serotypes 3, 37, and 8 represented a higher proportion of adult carriage than expected. Predicted carriage serotype distributions for ≥65 years aligned closest with the young adult carriage serotype distribution.

**Conclusions:**

Nasal wash technique is highly sensitive. For some serotypes carried by adults aged ≥65 years, other adults may be an important reservoir for transmission. Age groups such as older children should also be considered.

Invasive pneumococcal disease (IPD) is caused by infection with *Streptococcus pneumoniae* (pneumococcus) and includes pneumonia, septicemia, and meningitis. More than 100 serotypes of *S pneumoniae* (characterized by polysaccharide capsule differences) have been identified [1].

Vaccine-type IPD declined in the United Kingdom (UK) following the introduction of pneumococcal conjugate vaccines (PCVs) into the national childhood immunization program. PCV7, targeting 7 serotypes (4, 6B, 9V, 14, 18C, 19F, 23F), was introduced in 2006 and by 2009–2010 had reduced vaccine-type IPD in England and Wales by 98% in children aged <2 years and by 81% in adults aged ≥65 years [2]. However, several non-vaccine serotypes increased in frequency (“serotype replacement”) [2]. In 2010, PCV13, which targets an additional 6 serotypes (1, 3, 5, 6A, 7F, 19A), replaced PCV7 [3]. PCV13 led to a significant reduction in vaccine-type IPD among all ages and by 2016–2017, despite the increases in non-vaccine-type IPD, total IPD across all ages was 37% lower than the pre-PCV7 period [4].

Reductions in IPD have occurred via direct protection of vaccinated children and indirect protection of all age groups through the ability of PCVs to reduce vaccine-type carriage and hence transmission. However, reductions in PCV13 vaccine-type IPD have plateaued in adults since 2013–2014 [4], and PCV13 vaccine-type pneumonia remains common [5–7] despite vaccination of adults aged ≥65 years (∼70% coverage) with a pneumococcal polysaccharide vaccine (PPSV23) since 2005 [8].

PCV15 (containing PCV13 serotypes and serotypes 22F and 33F) and PCV20 (containing PCV15 serotypes and serotypes 8, 10A, 11A, 12F, and 15B) have been licensed for use in the UK, and in June 2023 the Joint Committee for Vaccination and Immunisation advised that either PCV20 or PPSV23 could be used for the adult pneumococcal program in the future [9–12].

The prevalent paradigm is that carriage occurs most commonly in young children and that transmission is almost entirely driven from this age cohort to other age cohorts [13]. How carriage in children relates to carriage in adults, and adult IPD, is less well understood. We aimed to compare carriage and IPD serotype distributions for children and adults in England and relate these to IPD and estimated carriage serotype distributions in older adults, to better understand adult carriage and potential benefits of higher-valency PCV introduction to adults.

## METHODS

### Sampling and Laboratory Methods

#### Carriage in Adults

Healthy volunteers aged ≥18 years were enrolled, with informed consent, in the experimental human pneumococcal carriage research program in the Liverpool School of Tropical Medicine, UK, 2011–2019. All 17 studies were approved by the local National Health Service Research Ethics Committee. Methods and inclusion/exclusion criteria have been previously reported [14]. All participants had no close contact with at-risk individuals during the study, including children aged <5 years, healthcare workers, patients with respiratory or immunosuppressive comorbidities, or those receiving steroid therapy (Supplementary Methods). Vaccination status was not ascertained but participants were not eligible for PCV (all born prior to 2002), though adults ≥65 years of age may have received PPSV23.

All volunteers underwent nasal wash (NW) screening for pneumococcal colonization at their first visit; all baseline results were included in this analysis. Methods for collection and the processing of NWs for bacteriological detection have been previously described [14, 15]. Pneumococcal isolates were serotyped by latex agglutination test and confirmed, when required, by the Senti-SP v1.6 molecular serotyping microarray (BUGS Bioscience, UK).

For negative-culture samples, bacterial genomic DNA was extracted from both raw and culture-enriched NW samples (Supplementary Methods).

#### Carriage in Children

Carriage data for children aged 13–48 months were combined from 2 cross-sectional studies in the Thames Valley, UK, carried out between February 2014 and August 2015 (988 children, 473 carriers [47.9% 572 isolates) and between June 2017 and August 2019 (795 children 413 carriers 51.8% 492 isolates) 16, 17]. Serotype-specific carriage was stable between these 2 time periods, apart from an increase in 7C [16]. All children included in these studies had received 3 doses of PCV13 according to the UK immunization schedule [18].

Nasopharyngeal swabs were collected and processed according to World Health Organization (WHO) guidelines [16, 19].

#### IPD in Adults and Children

IPD is characterized by detection of pneumococcus in a normally sterile site by culture or through polymerase chain reaction (PCR) in cerebrospinal or pleural fluid. Numbers of IPD cases in England aged 13–48 months, 18–44 years, and ≥65 years, for each serotype, were extracted from the national database for England maintained by the UK Health Security Agency (UKHSA) for 2014–2019. Serotyping of IPD isolates was performed by UKHSA [4].

#### Statistical Methods

Adult pneumococcal carriage prevalence in England and 95% confidence intervals (CIs) were calculated using a binomial exact method for the age groups 18–29 years, 30–44 years, and ≥45 years, and for males and females. An age- and sex-standardized estimate for England was calculated using 2015 (midpoint 2011–2019) Office for National Statistics population estimates [20].

Carriage geometric mean density measurements (in colony-forming units per milliliter [CFU/mL]) for adults were compared for PCV13 and non-PCV13 serotypes.

Carriage data for a subset of adults aged 18–44 years were compared with data for children aged 13–48 months and presented as the percentage of each serotype out of total isolates. “Excess” serotypes in adults and child serotype distributions were calculated by subtracting the percentage of each serotype found in child carriage from the percentage of each serotype found in adult carriage and vice versa. The age group 18–44 years was chosen to align with published data for IPD in England, and because the majority of our participants were young adults (Supplementary Figure 1).

Serotype-specific comparisons of carriage between children and adults were performed using Fisher exact test, reported as odds ratios (ORs, ratio of carriers/noncarriers for children/adults) with 99% CIs. *P* values <.01 were considered significant to allow for multiple comparisons.

IPD numbers were adjusted to account for the proportion of cases within each year that were not serotyped, assuming these cases had the same serotype distribution as those serotyped.

To enable comparisons of different numbers of isolates/cases for each age cohort, child and adult carriage and IPD serotype distributions were presented as the percentage of each serotype out of total isolates or IPD cases.

Serotype-specific case-carrier ratios for children and adults were calculated by dividing the average annual number of serotype-specific IPD cases in England in 2014–2019 by the estimated annual number of carriers of that serotype in England. The latter was calculated by multiplying study carriage prevalence of the serotype for 2014–2019 by the age-specific population of England in 2017 (midpoint). A measure of certainty in the estimates for each serotype was calculated by summing the numerators for carriage and IPD across children and adults (larger total numerators = greater certainty). A weighted (based on certainty) linear regression line was fitted and correlation coefficient determined from *R*^2^.

To calculate the predicted number of each serotype in carriage for adults aged ≥65 years, the number of IPD cases in adults ≥65 years for each serotype was divided by the case-carrier ratio calculated for children or young adults. Where case-carrier ratios were not available, the child case-carrier ratio could sometimes be estimated from the adult ratio (and vice versa) by using the fitted regression line relationship so that 33 serotypes were included in total. “Other” serotypes comprised 5% for children and 12.5% for adults, so a midpoint of 9% was used for age ≥65 years.

Predicted serotype distributions in carriage for adults aged ≥65 years were compared with actual 18–44 years and child carriage serotype distributions and the percentage agreement was calculated by subtracting the percentage in carriage in young adults or children for each serotype from the predicted percentage in carriage for ≥65 years. Positive differences were then summed and subtracted from 100.

Analyses were performed in R version 4.3.1 software.

## RESULTS

### Adult Carriage Population

#### Carriage Prevalence

Results were available for 1631 participants between 2011 and 2019 (Supplementary Tables 1 and 2), of whom 967 (59.3%) were female. Median age was 21 years (range, 18–80 years), reflecting a predominantly student population (Supplementary Figure 1).

*Streptococcus pneumoniae* was detected in of 97 of 1631 (5.9%) participants. England age- and sex-standardized prevalence was 6.4% (Table 1). Carriers ranged from 18 to 58 years of age. More than a single serotype was detected in only 1 participant, carrying serotypes 3 and 8.

**Table 1.** Adult Pneumococcal Carriage Prevalence by Age Group and Sex, Liverpool, England, 2011–2019, and Age and Sex Standardized to England 2015 Population

Age Group and Sex	No. of Participants	No. of Pneumococcal Carriers	Carriage Prevalence (95% CI)
18–29 y	1391	79	5.7 (4.5–7.0)
M	557	35	6.3 (4.4–8.6)
F	834	44	5.3 (3.9–7.0)
30–44 y	137	13	9.5 (5.1–15.7)
M	62	8	12.9 (5.7–23.9)
F	75	5	6.7 (2.2–14.9)
≥45 y	103	5	4.9 (1.6–11.0)
M	45	4	8.9 (2.5–21.2)
F	58	1	1.7 (.0–9.2)
Overall (≥18 y)	1631	97	5.9 (4.8–7.2)
M	664	47	7.1 (5.2–9.3)
F	967	50	5.2 (3.9–6.8)
Age- and sex-standardized England estimate			6.4 (2.5–14.6)
Age-standardized England estimate for males			9.4 (3.7–19.3)
Age-standardized England estimate for females			3.6 (1.3–10.2)

Abbreviations: CI, confidence interval; F, female; M, male.

#### Serotype Distribution

**Figure 1.**
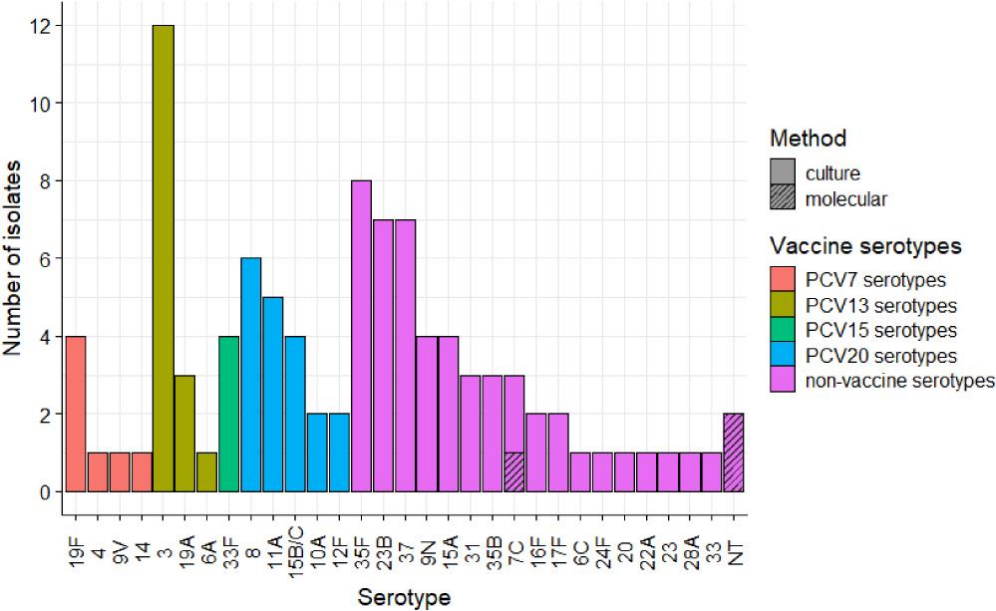
Serotypes detected in carriage in adults aged ≥18 years, 2011–2019, Liverpool, England (98 isolates from 97 adults). Abbreviations: NT, nontypeable; PCV, pneumococcal conjugate vaccine (number indicates valency).

In 2011–2019, 23 of 1631 adults aged ≥18 years (1.4% [95% CI, .9%–2.1%]) were carrying a PCV13 serotype, most commonly serotypes 3 (12/1631, 0.7% [95% CI .4%–1.3%]) and 19F (4/1631, 0.2% [95% CI, .1%–.6%]) (Figure 1). Of the 2 additional PCV15 serotypes (22F and 33F), only serotype 33F was detected (in 4 adults, 0.2%). A further 19 adults (1.2%) were carrying serotypes 8, 11A, 15B/C, 10A, and 12F contained in PCV20. The 5 most frequently isolated serotypes in our cohort were 3, 35F, 23B, 37, and 8 (Figure 1). Serotypes not contained in PCV20 were carried by 52 of 1631 adults (3.2% [95% CI, 2.4%–4.2%]).

In total, 94 (95 isolates) carriers were identified by culture, and 3 additional carriers (3 isolates; one 7C and 2 nontypeable) were identified by molecular methods on culture-negative samples (Figure 1).

#### Colony Density

Density measurements were available for 74 of 98 (76%) isolates during 2011–2019 (Supplementary Figure 2), of which 72 of 74 were from 2014 to 2019. There was no significant difference in geometric mean density overall between PCV13 serotypes (175 CFU/mL, n = 17) and non-PCV13 serotypes (53 CFU/mL, n = 57) (*P* = .26). Geometric mean density across all serotypes was 70 CFU/mL (median, 44 CFU/mL).

### Comparison With Carriage in Children (2014–2019)

#### Serotype Distribution

**Figure 2.**
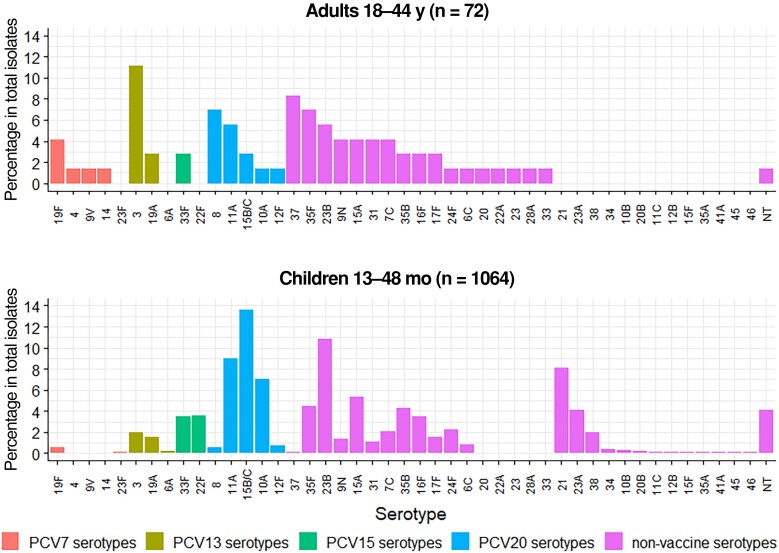
Comparison of adult (2014–2019) and children's (2014–2015 and 2017–2019) pneumococcal carriage serotype distributions. Abbreviations: NT, nontypeable; PCV, pneumococcal conjugate vaccine (number indicates valency).

In a subset of adults aged 18–44 years (n = 1289), 72 carriers (all single isolates) were identified (Supplementary Table 2). The adult (18–44 years) serotype distribution contained a higher proportion of PCV13 serotypes (16/72, 22.2%) compared with children (46/1064, 4.3%) (Figure 2). The most frequently isolated serotypes in adults were 3 (8 isolates, 11.1%), 37 (6 isolates, 8.3%), 8 and 35F (5 isolates each, 6.9%), and 11A and 23B (4 isolates each, 5.6%). In children, the most frequent serotypes were 15B/C (145 isolates, 13.6%), 23B (115 isolates, 10.8%), 11A (96 isolates, 9.0%), 21 (86 isolates, 8.1%), and 10A (75 isolates, 7.0%). Serotypes 3, 37, and 8 contributed 21 (2.0%), 1 (0.1%), and 6 (0.6%) isolates, respectively, in children.

**Figure 3.**
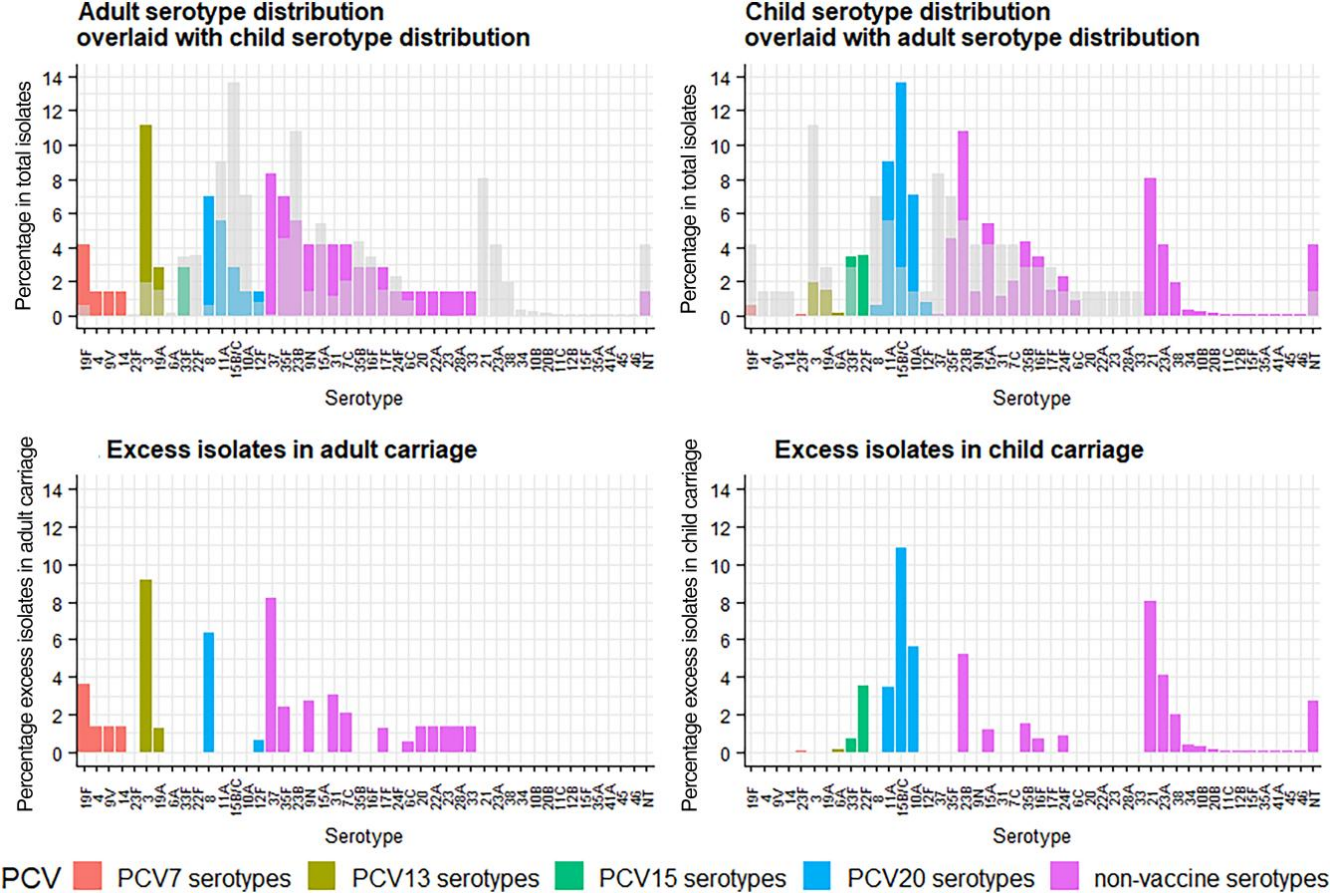
Adult (2014–2019) and children's (2014–2015 and 2017–2019) pneumococcal carriage serotype distributions overlaid (gray) and subtracted from one another to show excess isolates in each distribution. Abbreviations: NT, nontypeable; PCV, pneumococcal conjugate vaccine (number indicates valency).

Serotypes with the greatest “excess” isolates in adult carriage were 3, 37, and 8 (Figure 3*C*). Serotypes found in higher proportions in child compared with adult carriage were 15B/C, 21, 10A, and 23B (Figure 3*D*).

For serotypes detected in adults and children, the odds of carriage was not significantly different (*P* > .01) between adults and children for serotypes 37, 8, 19F, 3, 31, 12F, 6C, and 9N (remaining serotypes all greater odds of carriage in children) (Supplementary Figure 3, Supplementary Table 3).

#### Invasive Pneumococcal Disease

In total, 4237 IPD cases in adults aged 18–44 years (average annual incidence, 3.6 cases per 100 000) and 776 IPD cases in children aged 13–48 months (average annual incidence, 6.3 cases per 100 000) were reported in England between 2014 and 2019 inclusive, of which 3898 (92%) and 684 (88%), respectively, were serotyped.

The top 5 serotypes causing IPD in adults aged 18–44 years during 2014–2019 were 8 (1258 cases, 32.3% of those serotyped), 12F (642 cases, 16.5%), 9N (229 cases, 5.9%), 3 (227 cases, 5.8%), and 22F (189 cases, 4.8%); the top 5 serotypes causing IPD in children aged 13–48 months were 12F (100 cases, 14.6% of those serotyped), 15B/C (81 cases, 11.8%), 10A (68 cases, 9.9%), 23B (56 cases, 8.2%), and 24F (49 cases, 7.2%).

**Figure 4.**
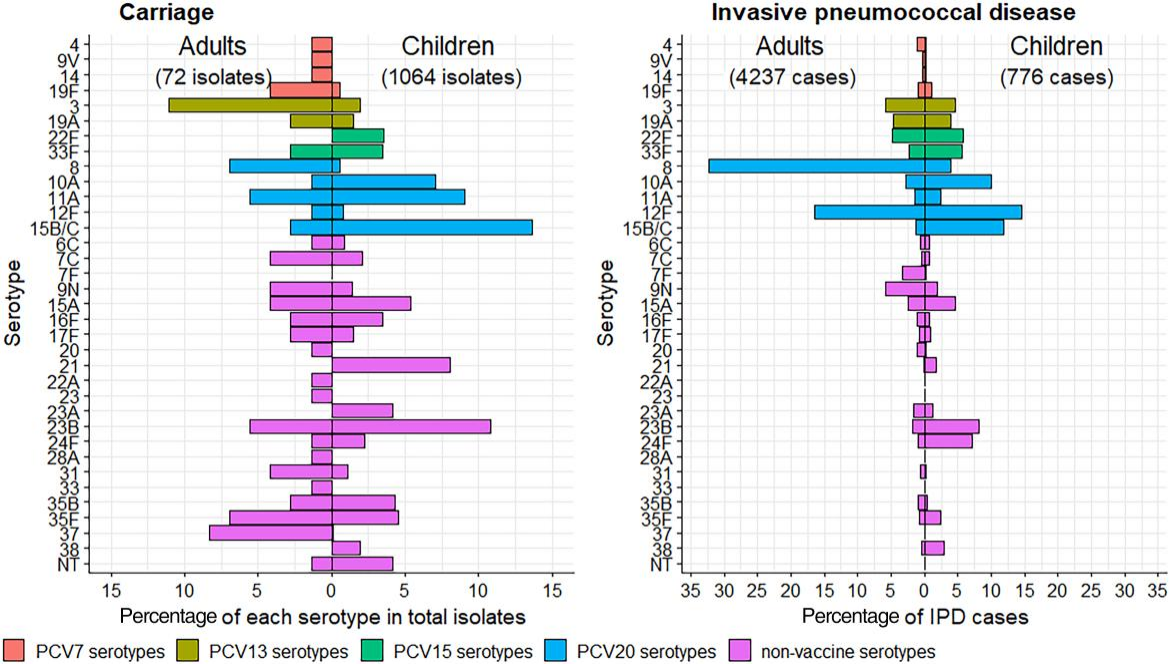
Comparison of child (13–48 months) and adult (18–44 years) carriage and invasive pneumococcal disease isolates, 2014–2019. To assist comparison of serotype distributions, this is for serotypes included ≥1% in carriage and/or disease for children and/or adults only. Serotypes are shown in numerical order within vaccine group. Abbreviations: IPD, invasive pneumococcal disease; NT, nontypeable; PCV, pneumococcal conjugate vaccine (number indicates valency).

**Figure 5.**
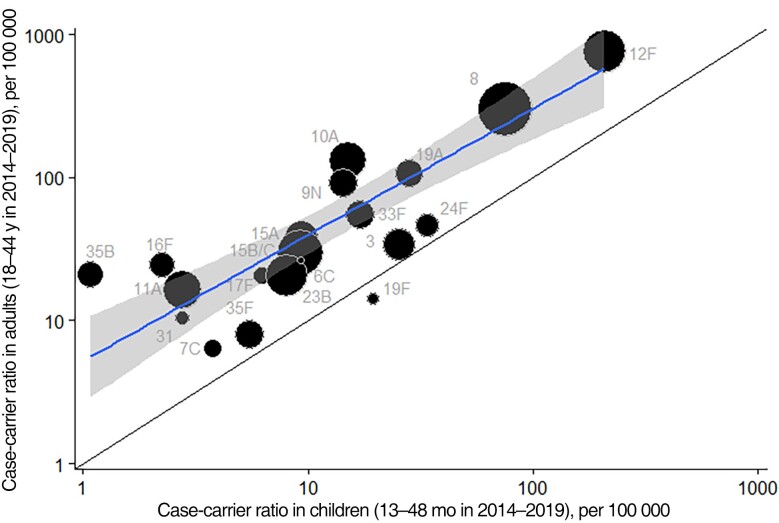
Case-carrier ratios per 100 000 population, children aged 13–48 months (2014–2019) vs adults aged 18–44 years (2014–2019), for serotypes with at least 1 case and carrier detected across both cohorts. Larger circles indicate more confidence. Line with shaded confidence intervals is weighted linear regression line: log_10_(y)= 0.7181 + 0.8820*log_10_(x), *R*  ^2^ = 0.8.

Despite differences in carriage and IPD serotype distributions between children and adults (Figure 4), case-carrier ratios for children and adults were highly correlated (*R* = 0.9) (Figure 5).

In general, serotypes with the highest case-carrier ratios caused most disease (Supplementary Figure 4*A–C*). The majority of case-carrier ratios (14/20) were not significantly different between adults and children (Supplementary Figure 4*A*). The remainder were higher in adults, and there was a general pattern of higher case-carrier ratios in adults compared with children (Supplementary Figure 4*A*, Figure 5).

**Figure 6.**
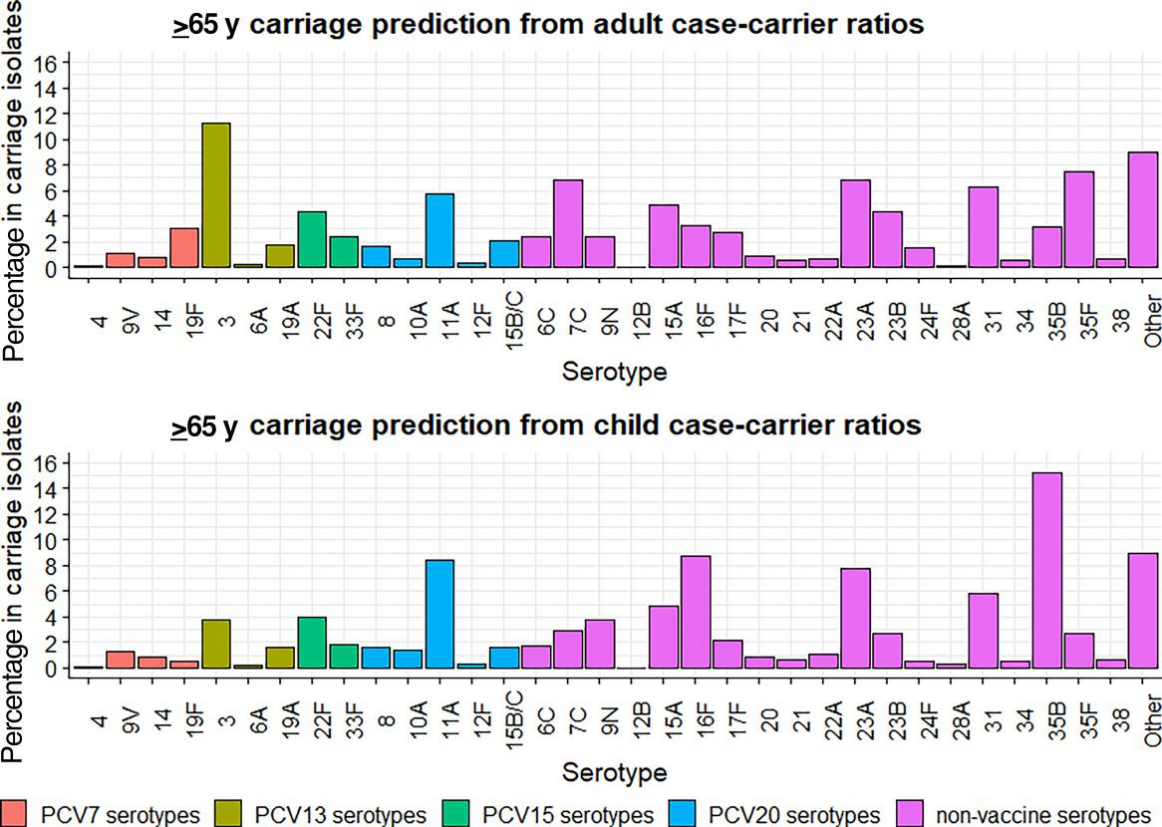
Age ≥65 years predicted carriage serotype distributions based on ≥65 years national invasive pneumococcal disease data, calculated with young adult (18–44 years) and child (13–48 months) case-carrier ratios. Abbreviation: PCV, pneumococcal conjugate vaccine (number indicates valency).

The predicted ≥65 years carriage serotype distribution based on the case-carrier ratios for adults aged 18–44 years (Figure 6*A*) showed 78.5% agreement with the young adult carriage serotype distribution and 59.4% agreement with the children's carriage serotype distribution (Supplementary Figure 5). The predicted ≥65 years carriage serotype distribution based on child case-carrier ratios (Figure 6*B*) showed 61.9% agreement with the young adult carriage serotype distribution and 58.8% agreement with the children's carriage serotype distribution (Supplementary Figure 6). The IPD serotype distribution in adults ≥65 years is provided for reference (Supplementary Figure 7).

## DISCUSSION

### Key Findings

We found a higher proportion of PCV13 serotypes (22.2%) in our young adult population compared with children (4.3%). The different serotype distribution in young adults compared with children, and particular abundance of serotypes 3, 37, and 8, suggests that these serotypes may be circulating within this adult population or among older children in whom the carriage serotype distribution is currently unknown.

Invasiveness was generally higher in young adults compared with children, and ratios were correlated. The close alignment of the predicted ≥65 years carriage serotype distributions with the young adult carriage serotype distribution suggests adult-to-adult transmission could be important, but does not rule out transmission occurring from younger and older children to adults, and between older adults themselves.

Our study demonstrates that molecular techniques only minimally improve the sensitivity of *S pneumoniae* detection when using NW to assess carriage. This contrasts with the substantial improvement in detection by molecular technique relative to classical microbiology using naso- or oropharyngeal swabs, the current WHO gold standard technique for carriage detection in adults [19, 21].

### Comparison to Other Studies

Previous studies of carriage in England post-PCV13 introduction have taken nasopharyngeal swabs within young-family households and found adult carriage prevalence of 2.8%–9% [22–24]. The similar carriage prevalence in our adult population, despite using the more sensitive NW technique, may reflect lack of contact with children. Previous studies in England have found proportions of vaccine serotypes 0–44% within adult populations, though limited by small numbers [22–24]. These estimates are point prevalences and longitudinal prevalence estimates in adults are rare [25]; any particular carriage study only samples a fraction of the upper airway, with variation depending on methodology; it is likely that even in sampled areas it is difficult to obtain pneumococci contained in biofilms, residing below the mucosal surface, or occurring at low density. Consequently, true carriage prevalences over time may be much higher than reported in the literature.

Serotype distributions for UK children aged 6–12 months and 12–48 months have previously been found to be similar [16]. However, different serotype carriage serotype distributions have been observed between younger (<24 months) and older (24–59 months) children in Israel, and the patterns in older children correlated better with patterns of IPD in adults (adult carriage data unavailable), supporting the idea of carriage serotype distributions changing between early childhood and adults [26]. Correlation between case-carrier ratios for adults and children, as well as higher ratios in adults compared with children, has previously been observed for Navajo Nation adults and children in the United States [27]. Serotype-specific invasiveness in children has previously been shown to be stable across different time periods and geographies [28]. Our invasiveness estimates correlate with recently published estimates by Løchen et al (Supplementary Figure 8*A* and 8*B*) [29]. Carriage prevalence in adults aged ≥65 years is estimated to be approximately 2%, but even if 6%, as seen in young adults, we would expect case-carrier ratios for this age group to be higher than for young adults given the higher numbers of IPD cases in adults aged ≥65 years [22, 30].

Carriage data for UK adults ≥65 years are not available in sufficient numbers to compare with our predicted profile [22], and comparisons with other countries are limited by differences in vaccination schedules and/or family/population structures. Alignment of the ≥65 years predicted serotype distributions with the young adult serotype distributions and suggestion of adult-to-adult transmission is supported by similar odds of carriage for children and adults for particular serotypes, and mixing matrices for the ≥65 years age group indicating higher contact rates with younger adults compared with children [31, 32]. Our young adult population did not have contact with young children, further suggesting acquisition of pneumococci from other age groups.

The observed abundance of serotype 3 and other PCV13 serotypes in adults indicates that these serotypes circulate within the unvaccinated adult population despite years of PCV13 use in children. Although serotype 37 was prominent in adult carriage, it was not reported in adult (or child) IPD for the same period, suggesting low invasiveness for this serotype currently. In contrast, serotype 8 is one of the main causes of IPD in adults in England. Recent modeling suggests that elimination of serotype 33F (included in both PCV15 and PCV20) could result in serotype switching to serotypes 11A and 8 (both included in PCV20 but not PCV15) [33], which could potentially increase the circulation of the latter if a vaccine is used that contains 33F but not 8 and 11A. The presence and possible transmission of serotype 8 among adults suggests that introduction of PCV20 only in children may have less impact than might otherwise be expected and that more substantial reductions may require direct immunization of adults.

In a cross-sectional study in 2010–2011, serotype 22F was the most frequently detected serotype in 3 of 9 colonized UK parents, followed by serotypes 3 and 19A (2 isolates each) [22]. Serotype 22F was not detected in our adult cohort, but it was detected in children. In a 2014–2015 UK carriage study, the most frequently isolated serotypes from parents were 21, 23B, and 38 (each found in 3 of 21 colonized parents), of which only serotype 23B was detected in our young adult study population (all 3 serotypes were isolated from our child comparison population [17]). These serotypes could mainly be transmitted from children to adults.

Our adult study population was predominantly students, a population group with many social contacts in crowded settings, providing multiple opportunities for transmission. Young adults are a reservoir of infection for *Neisseria meningitidis* [34, 35], and carriage prevalence increases as attendance at university/college progresses [36]. We did not have sufficient numbers to explore this, but it is plausible that this population group could be a reservoir for the serotypes found at particular abundance within young adult carriage. A longitudinal study of adults aged 25–50 years in Portugal found that approximately 20% of the adults were intermittent carriers and 10% were persistent carriers (>4 months) and suggested some adults may act as reservoirs of pneumococci [37].

Similar colony density in adults for PCV13 and non-vaccine serotypes contrasts with the finding in PCV13-vaccinated children [16]. Density measurements cannot be directly compared between children and adults because of different methodology, but the lower orders of magnitude for adult measurements suggest lower colony density in adults.

### Strengths and Limitations

The IPD data relate to the national population and for adults will likely have tended toward older individuals within the 18–44 years age band and those with underlying comorbidities, unlike our young, healthy adult study population. Crucially, also our study population was not exposed to children. However, although the study population met specific exclusion criteria, they may have mixed with others who did not meet those criteria, or had contact with risk groups prior to enrollment, meaning serotypes with long durations of carriage would have been unaffected.

Different methods were used to sample adults and children, and the serotype identification methodology differed, though there was some overlap.

The adult and child populations were from different geographical areas of England, so we cannot exclude regional differences in circulating serotypes; however, vaccination coverage of the PCV booster dose at age 2 years was similar across both study regions [38].

The ≥65 years predicted carriage serotype distributions are approximate and limited to the 33 serotypes with available case-carrier ratios. The case-carrier ratios have different sized CIs; this uncertainty was not incorporated into the predicted serotype distributions. The assumption that “other” serotypes comprise around 9% of ≥65 years carriage was chosen to try to minimize bias toward either the adult or child serotype distribution, but this value is unknown. Serotype 37 was prominent in the young adult serotype distribution but could not be incorporated into the older adult serotype distribution because its case-carrier ratio could not be calculated (zero IPD cases).

We did not calculate predicted serotype distributions in carriage for older children because these would be based on relatively low IPD case numbers [39].

## CONCLUSIONS

Our finding of a distinct adult pneumococcal carriage serotype distribution gives an important insight into overall and serotype-specific carriage and disease dynamics. The identification of particular serotypes that may be circulating in adults, and the similarity of the predicted serotype distributions for older adults to the serotype distribution in young adults, suggests that this group of adults could be a reservoir for pneumococcus in the community. With advancements in molecular methods for detection, future studies should investigate carriage and serotype distributions in adults ≥65 years of age. Further studies are needed to better understand the role of adults and older children as transmitters of pneumococcus, particularly associated with increased nasal colonization density driven by upper respiratory tract virus coinfections [27, 40, 41]. This has implications for the optimal use of vaccines against pneumococcus and respiratory viruses (eg, respiratory syncytial virus, influenza) to provide increased protection for adults at risk, including the elderly.

## Supplementary Data

Supplementary materials are available at *The Journal of Infectious Diseases* online (http://jid.oxfordjournals.org/). Supplementary materials consist of data provided by the author that are published to benefit the reader. The posted materials are not copyedited. The contents of all supplementary data are the sole responsibility of the authors. Questions or messages regarding errors should be addressed to the author.

## Supplementary Material

jiae351_Supplementary_Data

## Notes

***Acknowledgments.*** The authors express their gratitude to all participating adults and children.

***Author contributions.*** Study concept and design: D. E. S., D. M. F., and K. S. T. Recruiting and consent of participants: A. C., R. C.-J., R. K., E. P., H. Ra., H. Ro., and A. H.-W. Data collection and management: P. A., M. B., R. C.-J., D. E. S., L. H., R. K., E. P., H. Ra., and H. Ro. Sample processing: J. B., D. E. S., N. K. F., K. G., A. H., E. H., F. E., J. H., L. H., D. L., E. M., S. P., M. R., C. S., and A. M. Statistical planning and analysis: K. S. T. and M. V. Data analysis and interpretation: E. B., M. B., D. E. S., D. M. F., N. K. F., B. G., D. L., E. M., A. H., L. H., A. J. P., S. P., C. S., C. T., K. S. T., and M. V. All authors read and approved the manuscript.

***Ethics.*** The UK Health Security Agency has legal permission to process confidential information for national surveillance of communicable diseases without individual patient consent (Regulation 3 of Health Service Regulations 2002) and, as such, ethics committee approval was not required for the collection of IPD data.

***Data availability.*** Adult and child pneumococcal carriage data are available in the Supplementary Material. UKHSA collects data as part of national surveillance, so requests for invasive pneumococcal disease data can be made to UKHSA directly.

***Financial support*.** This work was conducted as a collaboration between Liverpool School of Tropical Medicine, University of Oxford, UK Health Security Agency, and Pfizer. The analysis of samples from the Liverpool carriage studies was funded by a collaborator award from Pfizer. The Oxford Vaccine Group children's carriage studies were investigator-initiated studies funded by Pfizer and sponsored by the University of Oxford. The research was supported by the National Institute for Health and Care Research (NIHR), Oxford Biomedical Research Centre and NIHR Clinical Research Network, Thames Valley and South Midlands. Funding to pay the Open Access publication charges for this article was provided through the JIS24001 - UK Institutions (Jisc affiliated) Hybrid 2024 collection Open Access Agreement.
