# Supplementary material for: Pneumococcal Carriage and Disease in Adults in England, 2011–2019: The Importance of Adults as a Reservoir for Pneumococcus in Communities
Source: J Infect Dis. 2024 Jul 16;231(1):e17–27. doi: 10.1093/infdis/jiae351 (PMC11793058; doi:10.1093/infdis/jiae351)
Supplement: jiae351_Supplementary_Data [file jiae351_supplementary_data.zip › Supplementary_material_22_May_2024.docx]

**Supplementary material: Pneumococcal carriage and disease adults in England 2011-2019: the importance of adults as a reservoir for pneumococcus in communities**

**Supplementary methods**

**Study exclusion criteria**

All of the adult studies except for one (the over 50 challenge study) excluded persons with “close physical contact with at risk individuals including elderly”.

The definition of “close contact” was left to the study team to interpret but would have excluded those living with and/or caring for elderly persons.

**DNA extraction and culture enrichment for pneumococcus detection**

On the day of extraction, the original raw bacterial pellet suspended in 800μl of STGG was thawed at room temperature for 20 mins and subjected to vigorous vortex. DNA extraction was performed using 200μl of NW. In addition, for the culture enrichment step, 50μl of raw NW pellet was cultured onto Columbia 5% sheep blood agar with 80μl gentamicin (1mg/ml). The plates were then incubated overnight, and the resulting bacterial growth was then harvested into 2ml of STGG, homogenized through vigorous vortexing. Two hundred microliters of culture-enriched NW were used for DNA extraction by the magnetic-bead technology of KingFisher with QIAamp DNA minikit (Qiagen, Manchester, UK) according to the manufacturer’s instructions. The final DNA extract was eluted into 100μl. The molecular detection of pneumococcus was conducted using a sequential single-plex quantitative qPCR that began with partial amplification of the *lytA* gene [1] followed by the *piaB* gene [2]. PCR mixtures, thermal cycling conditions, and qPCR bacterial quantification were conducted as previously described [3]. Samples were tested in duplicate, and a positive result was defined as both qPCR tests being positive with a cycle threshold (Ct) <40. A negative DNA extraction control (parallel extraction from sample buffer only), a qPCR negative control (master mix only), a qPCR positive control (pneumococcal Spn23F strain), and duplicates of each sample were amplified. Molecularly positive samples were serotyped using microarray (BUGS Bioscience) after re-culturing 50-100μl of the raw bacterial pellet on Streptococcal selective culture plates (COBA agar) overnight. The pneumococcal presumptive colonies were then subjected to DNA extraction prior to molecular serotyping.

**Supplementary figures and tables**

| **Year** | **Number of participants** | **Number of carriers** | **Per cent carriage** |
| --- | --- | --- | --- |
| 2011 | 68 | 5 | 7.4% |
| 2012 | 144 | 15 | 10.4% |
| 2013 | 32 | 1 | 3.1% |
| 2014 | 71 | 5 | 7.0% |
| 2015 | 163 | 9 | 5.5% |
| 2016 | 222 | 15 | 6.8% |
| 2017 | 349 | 15 | 4.3% |
| 2018 | 232 | 15 | 6.5% |
| 2019 | 350 | 17 | 4.9% |
| **Total** | **1631** | **97** | **5.9%** |

**Supplementary table 1: Number of participants and percentage of pneumococcal carriers per year (2011-2019)**

**Supplementary table 2. Carriage prevalence for different age groups and time periods**

| Age (years) | 2011-2019 | | | 2014-2019 | | |
| --- | --- | --- | --- | --- | --- | --- |
|  | Number of participants | Number of carriers | Carriage prevalence (95% confidence intervals) | Number of participants | Number of carriers | Carriage prevalence (95% confidence intervals) |
| 18+ | 1631 | 97 | 5.9 (4.8, 7.2) | 1387 | 76 | 5.5 (4.3, 6.8) |
| 18-44 | 1528 | 92 | 6.0 (4.9, 7.3) | 1289 | 72 | 5.6 (4.4, 6.9) |
| 18-64 | 1601 | 97 | 6.1 (4.9, 7.3) | 1357 | 76 | 5.6 (4.4, 7.0) |
| 65+ | 30 | 0 | 0.0 (0.0, 11.6) | 30 | 0 | 0.0 (0.0, 11.6) |


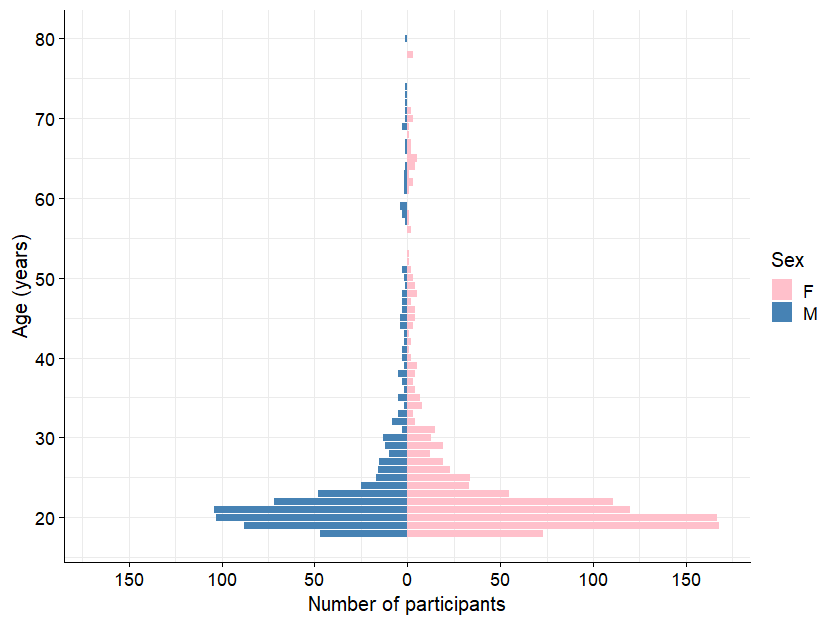


**Supplementary figure 1. Age and sex distribution of adult participants 2011-2019, Liverpool, England**


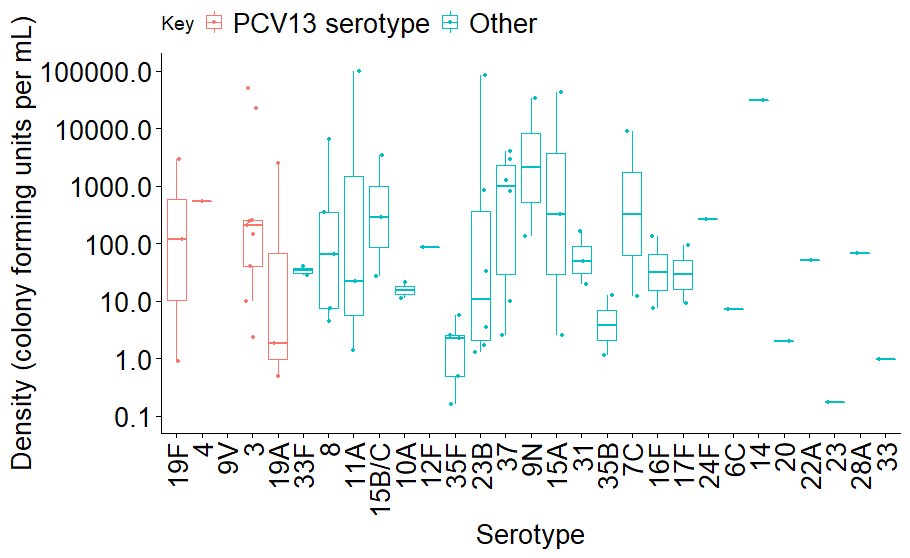


**Supplementary figure 2. Serotype densities in adults aged 18+ years 2011-2019, Liverpool, England (74 isolates with density measurements available)** PCV13=13-valent pneumococcal conjugate vaccine


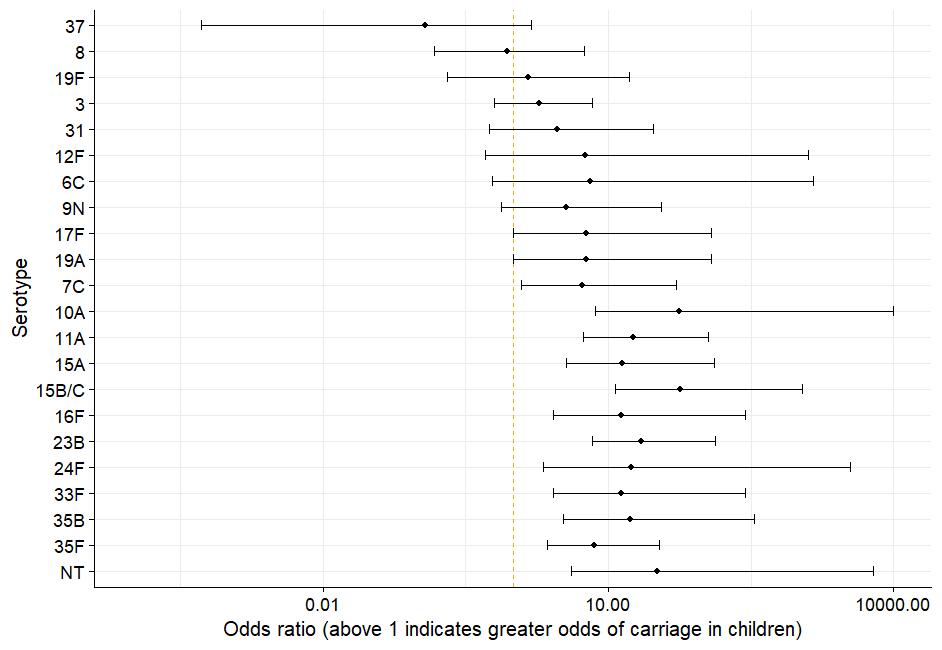


**Supplementary figure 3. Serotype-specific carriage odds ratios for children (13-48 months, Thames Valley, 2014/15 and 2017/19) vs adult (18-44 years, Liverpool, 2014-2019), with 99% confidence intervals.** NT=non-typable

| **Serotype** | **Number of child carriers** | **Prevalence in children (%)** | **Number of adult carriers** | **Prevalence in adults (%)** | **OR (99% confidence interval)** | **p-value** |
| --- | --- | --- | --- | --- | --- | --- |
| 37 | 1 | 0.1 | 6 | 0.5 | 0.1 (0.0, 1.6) | 0.024 |
| 8 | 6 | 0.3 | 5 | 0.4 | 0.9 (0.1, 5.6) | 1.000 |
| 19F | 6 | 0.3 | 3 | 0.2 | 1.4 (0.2, 16.7) | 0.742 |
| 3 | 21 | 1.2 | 8 | 0.6 | 1.9 (0.6, 6.8) | 0.132 |
| 31 | 12 | 0.7 | 3 | 0.2 | 2.9 (0.6, 29.7) | 0.115 |
| 12F | 8 | 0.4 | 1 | 0.1 | 5.8 (0.5, 1283.5) | 0.089 |
| 6C | 9 | 0.5 | 1 | 0.1 | 6.5 (0.6, 1424.4) | 0.052 |
| 9N | 15 | 0.8 | 3 | 0.2 | 3.6 (0.8, 36.3) | 0.031 |
| 17F | 16 | 0.9 | 2 | 0.2 | 5.8 (1.0, 122.5) | **0.007** |
| 19A | 16 | 0.9 | 2 | 0.2 | 5.8 (1.0, 122.5) | **0.007** |
| 7C | 22 | 1.2 | 3 | 0.2 | 5.4 (1.2, 51.6) | **0.002** |
| 10A | 75 | 4.2 | 1 | 0.1 | 56.5 (7.3, 9955.1) | **<0.001** |
| 11A | 96 | 5.4 | 4 | 0.3 | 18.3 (5.5, 111.7) | **<0.001** |
| 15A | 57 | 3.2 | 3 | 0.2 | 14.2 (3.6, 130.3) | **<0.001** |
| 15B/C | 145 | 8.1 | 2 | 0.2 | 56.9 (11.9, 1096.7) | **<0.001** |
| 16F | 37 | 2.1 | 2 | 0.2 | 13.6 (2.7, 273.8) | **<0.001** |
| 23B | 115 | 6.4 | 4 | 0.3 | 22.1 (6.7, 135.1) | **<0.001** |
| 24F | 24 | 1.3 | 1 | 0.1 | 17.6 (2.1, 3494.9) | **<0.001** |
| 33F | 37 | 2.1 | 2 | 0.2 | 13.6 (2.7, 273.8) | **<0.001** |
| 35B | 46 | 2.6 | 2 | 0.2 | 17.0 (3.4, 340.8) | **<0.001** |
| 35F | 48 | 2.7 | 5 | 0.4 | 7.1 (2.3, 34.7) | **<0.001** |
| NT | 44 | 2.5 | 1 | 0.1 | 32.6 (4.1, 6133.4) | **<0.001** |

**Supplementary table 3. Serotype-specific carriage odds ratios for children (13-48 months, Thames Valley, 2014/15 and 2017/19, N=1,783) vs adult (18-44 years, Liverpool, 2014-2019, N=1,289), with 99% confidence intervals.** P-values <0.01 in **bold**. NT=non-typable

Footnote: serotypes with no isolates detected in adults aged 18-44 years but significantly higher odds of detection in children (p<0.01) were: 22F (38 isolates, 2.1% prevalence in children), 21 (86 isolates, 4.8% prevalence in children), 23A (44 isolates, 2.5% prevalence in children), 38 (21 isolates, 1.2% prevalence in children). All other serotypes had no isolates detected in either adults or children and too few isolates detected to observe any significant differences between adults and children.


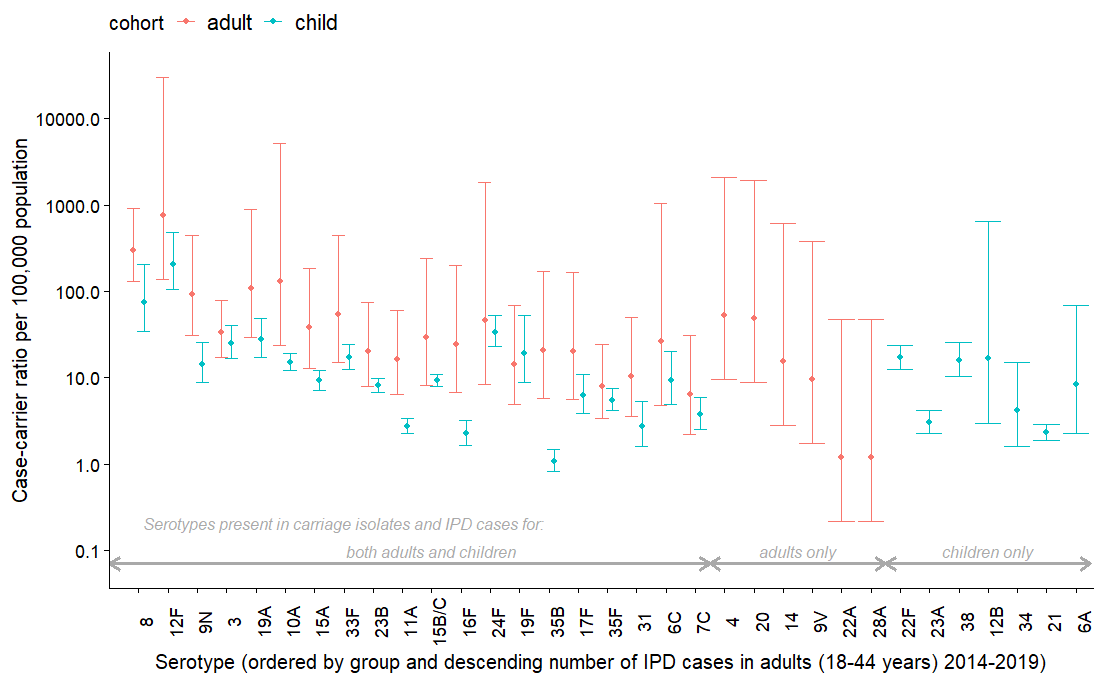


**Supplementary figure 4a. Case-carrier ratios with 95% confidence intervals for adults (18-44 years, 2014-2019) and children (13-48 months, 2014-2019) for serotypes present in carriage isolates and invasive pneumococcal disease (IPD) cases, England**

Footnote: serotypes are ordered by descending number of IPD cases in adults (18-44 years) 2014-2019


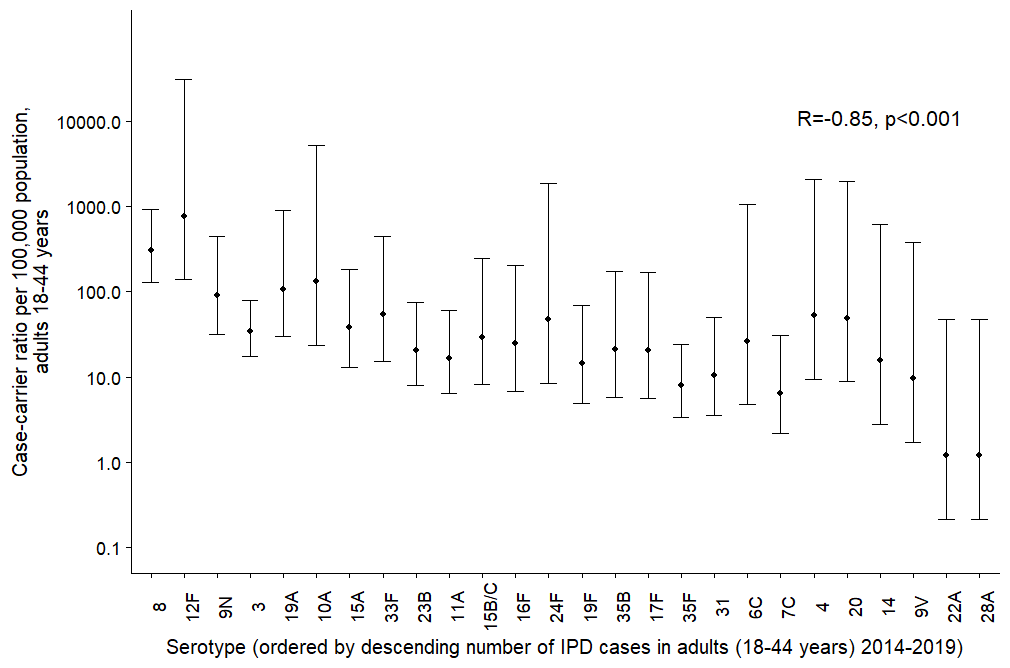


**Supplementary figure 4b. Case-carrier ratios with 95% confidence intervals for adults (18-44 years, 2014-2019) for serotypes present in carriage isolates and invasive pneumococcal disease (IPD) cases, England.**

Footnote: serotypes are ordered by descending number of IPD cases, R indicates Spearman’s correlation coefficient (based on rank order)


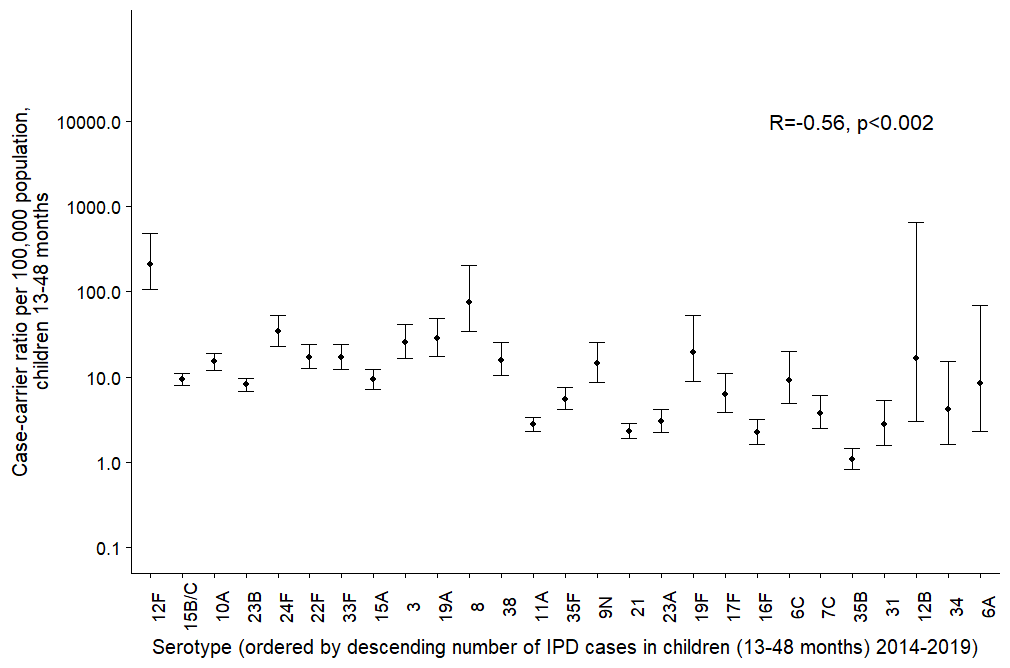


**Supplementary figure 4c. Case-carrier ratios with 95% confidence intervals for children (13-48 months, 2014-2019) for serotypes present in carriage isolates and invasive pneumococcal disease (IPD) cases, England.**

Footnote: serotypes are ordered by descending number of IPD cases, R indicates Spearman’s correlation coefficient (based on rank order)


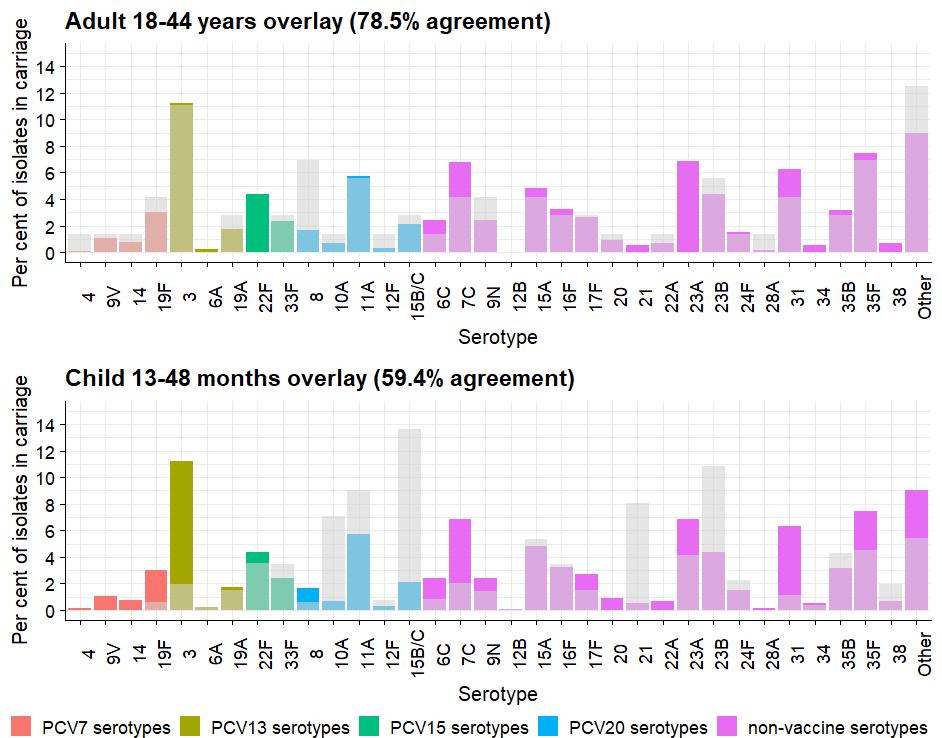


**Supplementary figure 5. Predicted 65+ years carriage serotype distribution calculated from young adult case-carrier ratios overlaid (grey shading) with A. young adult (18-44 years) and B. child (13-48 months) serotype distributions.** The percentage agreement indicates the total overlap between the predicted serotype distribution and the overlaid serotype distribution, i.e. the percentage of the predicted serotype distribution that is covered by the grey overlay.


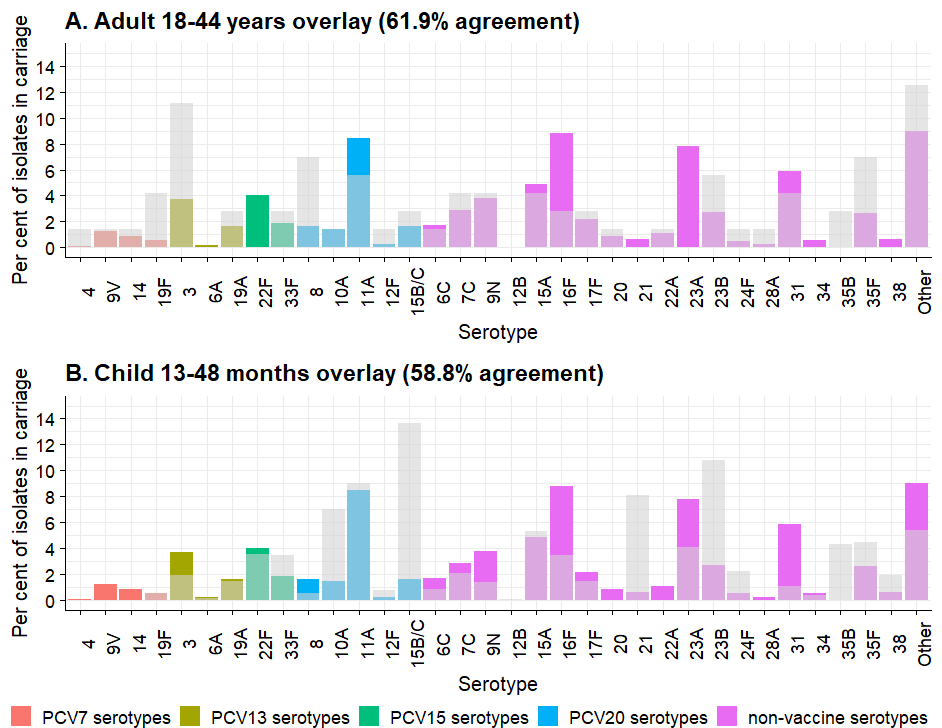


**Supplementary figure 6. Predicted 65+ years carriage serotype distribution calculated from child case-carrier ratios overlaid (grey shading) with A. young adult (18-44 years) and B. child (13-48 months) serotype distributions.** The percentage agreement indicates the total overlap between the predicted serotype distribution and the overlaid serotype distribution, i.e. the percentage of the predicted serotype distribution that is covered by the grey overlay.


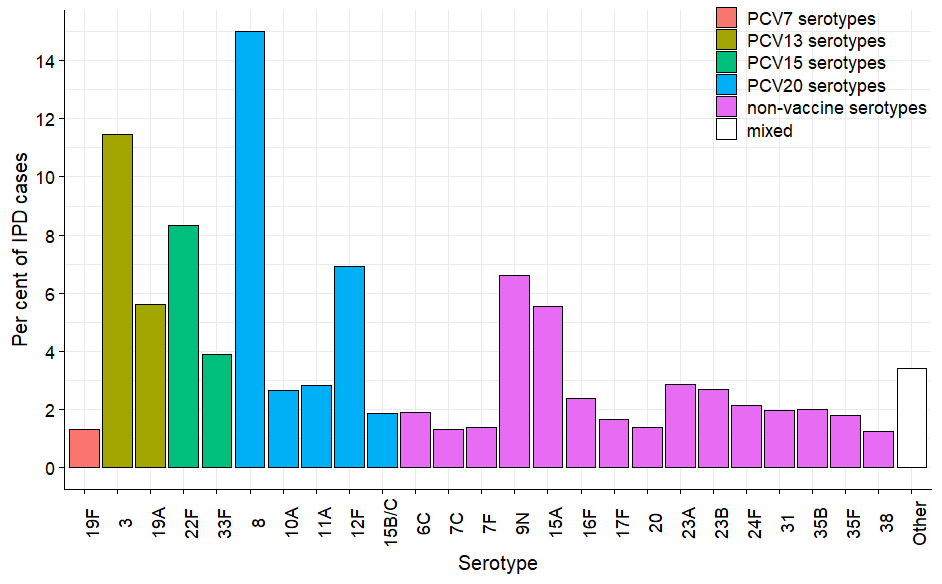


**Supplementary figure 7. Invasive pneumococcal disease serotype distribution for adults aged 65+ years, England, 2014-2019.** Figure displays all serotypes comprising ≥1% in IPD for this age group; serotypes comprising <1% are included in the ‘Other’ category.


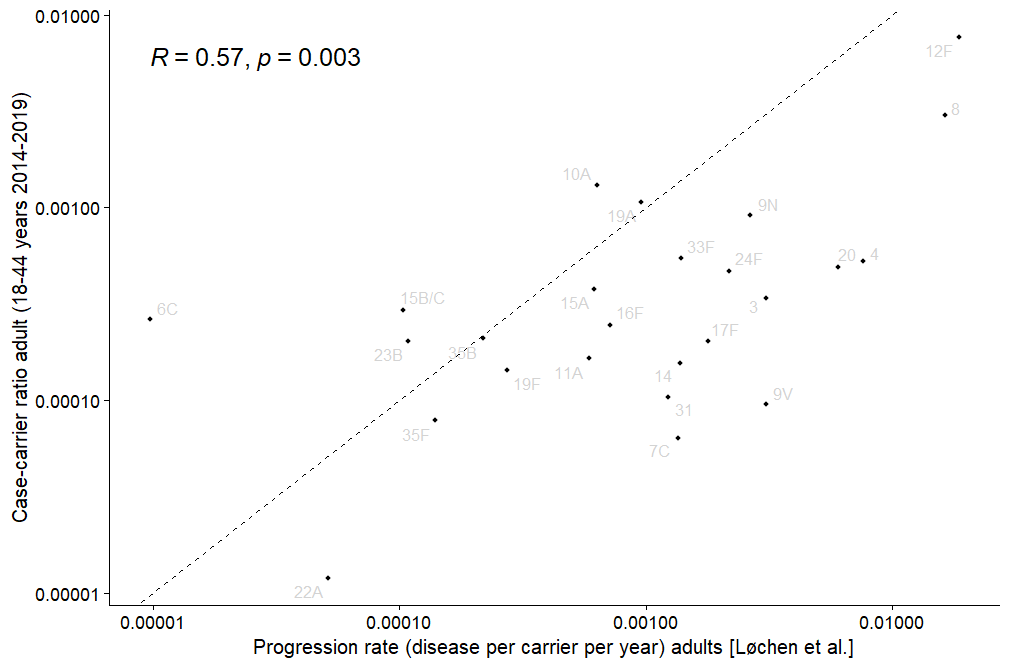


**Supplementary figure 8a. Comparison between case-carrier ratios for adults calculated in this study and the median estimates reported by Løchen et al. [S2 Dataset]** [4]**.** R indicates Pearson’s correlation coefficient. Dashed line y=x

Note: the ages of adults in the studies included by Løchen et al are mainly 18+ years as opposed to our more limited 18-44 years group so we would expect more variability in these estimates compared with our young adult population.


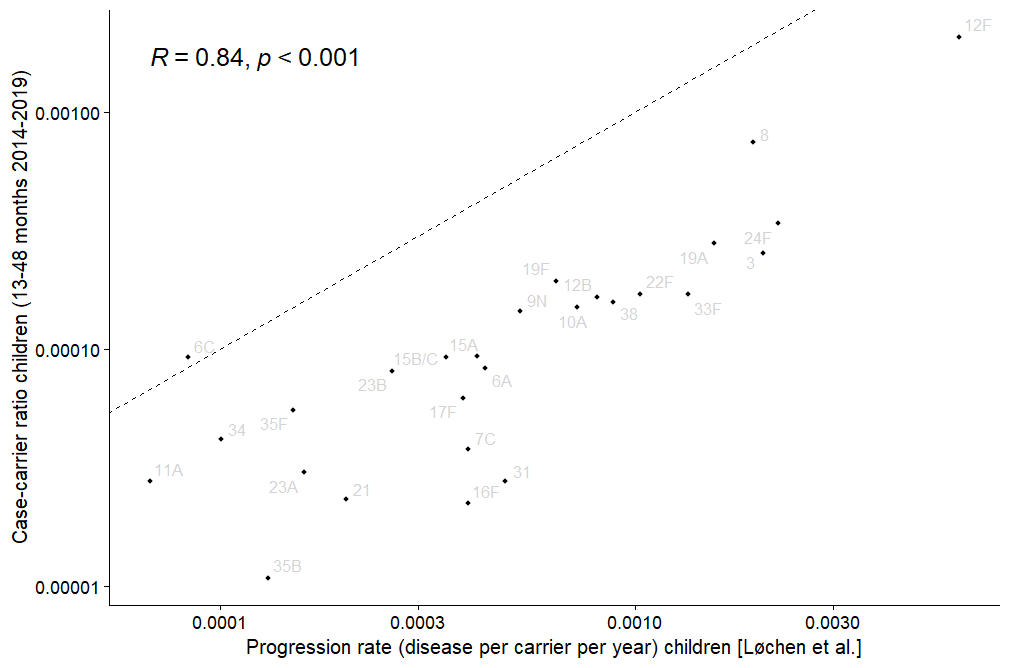


**Supplementary figure 8b. Comparison between case-carrier ratios for children calculated in this study and the median estimates reported by Løchen et al. [S1 Dataset]** [4]**.** R indicates Pearson’s correlation coefficient. Dashed line y=x

Note: the ages of children included in the studies included by Løchen et al range from <2 years to <18 years.

References

1. Carvalho M da GS, Tondella ML, McCaustland K, et al. Evaluation and improvement of real-time PCR assays targeting lytA, ply, and psaA genes for detection of pneumococcal DNA. J Clin Microbiol. **2007**; 45(8):2460–2466.

2. Trzciński K, Bogaert D, Wyllie A, et al. Superiority of Trans-Oral over Trans-Nasal Sampling in Detecting Streptococcus pneumoniae Colonization in Adults. PLoS ONE. **2013**; 8(3):e60520.

3. Mitsi E, Reiné J, Urban BC, et al. Streptococcus pneumoniae colonization associates with impaired adaptive immune responses against SARS-CoV-2. J Clin Invest. **2022**; 132(7):e157124.

4. Løchen A, Truscott JE, Croucher NJ. Analysing pneumococcal invasiveness using Bayesian models of pathogen progression rates. PLoS Comput Biol. **2022**; 18(2):e1009389.
